# Association of the classification and severity of heart failure with the incidence of contrast-induced acute kidney injury

**DOI:** 10.1038/s41598-021-94910-1

**Published:** 2021-07-28

**Authors:** Tian Xu, Maoning Lin, Xiaohua Shen, Min Wang, Wenjuan Zhang, Liding Zhao, Duanbin Li, Yi Luan, Wenbin Zhang

**Affiliations:** 1 Department of Cardiology, Key Laboratory of Cardiovascular Intervention and Regenerative Medicine of Zhejiang Province, Sir Run Run Shaw Hospital, School of Medicine, Zhejiang University, 3 East Qingchun Road, Hangzhou, Zhejiang Province 310016 People’s Republic of China; 2 Key Laboratory of Cardiovascular Intervention and Regenerative Medicine of Zhejiang Province, Hangzhou, 310016 China; 3 Department of Information Technology, Sir Run Run Shaw Hospital, School of Medicine, Zhejiang University, Hangzhou, 310016 China

**Keywords:** Interventional cardiology, Nephrology, Risk factors

## Abstract

Congestive heart failure (HF) is a known risk factor of contrast-induced acute kidney injury (CI-AKI). However, the relationship of the classification and severity of HF with CI-AKI remains under-explored. From January 2009 to April 2019, we recruited patients undergoing elective PCI who had complete pre- and post-operative creatinine data. According to the levels of ejection fraction (EF), HF was classified as HF with reduced EF (HFrEF) [EF < 40%], HF with mid-range EF (HFmrEF) [EF 40–49%] and HF with preserved EF (HFpEF) [EF ≥ 50%]. CI-AKI was defined as an increase of either 25% or 0.5 mg/dL (44.2 μmoI/L) in serum baseline creatinine level within 72 h following the administration of the contrast agent. A total of 3848 patients were included in the study; mean age 67 years old, 33.9% females, 48.1% with HF, and 16.9% with CI-AKI. In multivariate logistic regression analysis, HF was an independent risk factor for CI-AKI (OR 1.316, *p* value < 0.05). Among patients with HF, decreased levels of EF (OR 0.985, *p* value < 0.05) and elevated levels of N-terminal pro b-type natriuretic peptide (NT-proBNP) (OR 1.168, *p* value < 0.05) were risk factors for CI-AKI. These results were consistent in subgroup analysis. Patients with HFrEF were more likely to develop CI-AKI than those with HFmrEF or HFpEF (OR 0.852, *p* value = 0.031). Additionally, lower levels of EF were risk factors for CI-AKI in the HFrEF and HFmrEF groups, but not in the HFpEF group. NT-proBNP was an independent risk factor for CI-AKI in the HFrEF, HFmrEF and HFpEF groups. Elevated levels of NT-proBNP are independent risk factors for CI-AKI irrespective of the classification of HF. Lower levels of EF were risk factors for CI-AKI in the HFrEF and HFmrEF groups, but not in the HFpEF group.

## Introduction

Coronary angiography (CAG) and percutaneous coronary intervention (PCI) have become increasingly and widely applied in the diagnosis and treatment of coronary artery disease^1^. Concurrently, contrast-induced acute kidney injury (CI-AKI) has become a major complication of these procedures^2^. There is ample evidence indicating of increase in the CI-AKI incidence in patients undergoing cardiac catheterization procedures over the past decades, especially among patients with severe cardiovascular diseases. This was accompanied by prolonged hospitalization stay leading to a higher health cost and increased mortality^3–5^. The economic and health burdens of CA-AKI resulted in much interest in the prevention and treatment of CI-AKI^6^. Identifying the underlying risk factors and their associated biological pathways would allow targeting those at high risk^7^. Several risk factors and predictors for CI-AKI have been identified, such as elective use of intra aortic balloon pump (IABP), advanced congestive heart failure (HF), impaired renal function, elderly, anemia, diabetes mellitus, and increasing contrast media volume^8,9^.

Patients with HF are more prone to have comorbidities^10^, and the coexistence of CI-AKI with HF has been repeatedly reported^11–13^. The 2016 European Society of Cardiology (ESC) guidelines for HF classified HF into three groups based on ejection fraction (EF); HF with preserved EF (HFpEF), HF with reduced EF (HFrEF), and HF with mid-range EF (HFmrEF)^14^. N-terminal pro b-type natriuretic peptide (NT-proBNP) has also been used extensively to monitor the severity of cardiac dysfunction in patients with HF^15^. A previous study showed no significant difference in the incidence of CI-AKI among the three types of HF^16^. However, that was a single-center study with a limited sample size^16^.

Therefore, this multicenter study aimed to verify whether HF is an independent risk factor of CI-AKI, then explored the effect of the classification and severity of HF on the incidence of CI-AKI.

## Materials and methods

This multicenter retrospective study was conducted at Sir Run Run Shaw Hospital and its medical consortium hospitals. The study was conducted according to the criteria set by the Declaration of Helsinki involving experimenting on human subjects, and was approved by the ethics committee of Sir Run Run Shaw Hospital (NO.20201217-36). Informed consent was obtained from all participants.

### Population and procedures

**Figure 1:**
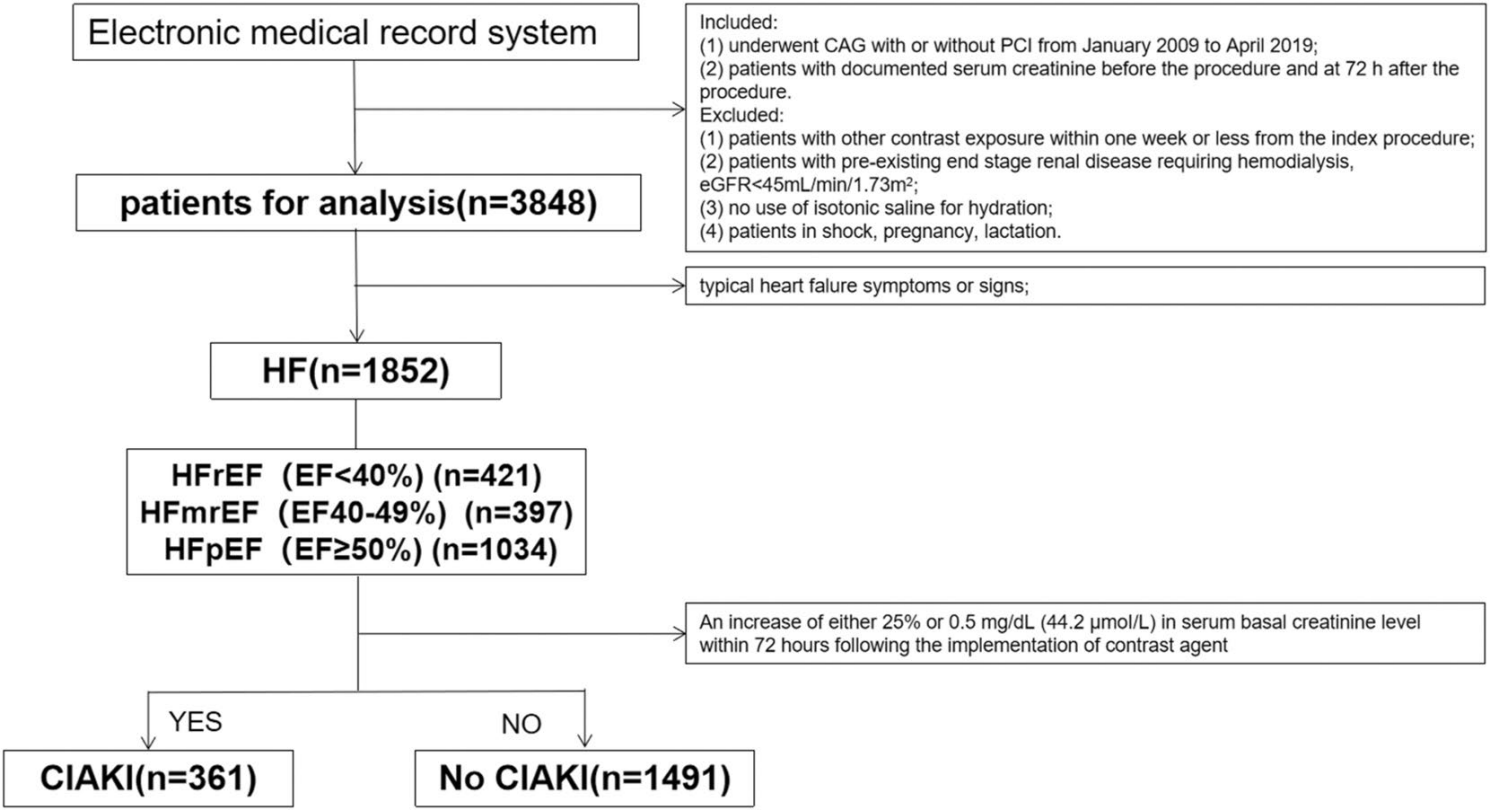
Flow chart for study design. *CAG* coronary angiography; *PCI* percutaneous coronary intervention; *eGFR* estimated glomerular filtration rate; *HF* heart failure; *EF* ejection fraction; *HFrEF* HF with reduced EF; *HFmrEF* HF with mid-range EF; *HFpEF* HF with preserved EF; *CI-AKI* contrast-induced acute kidney injury.

We included consecutive eligible patients who underwent CAG with or without PCI at Sir Run Run Shaw Hospital and its medical consortium hospitals from January 2009 to April 2019. Further details about the study population and recruitment are provided in Fig. 1.

The inclusion criteria were: (1) patients who had CAG with or without PCI; (2) patients with documented serum creatinine before and 72 h after the procedure. Exclusion criteria were: (1) patients with other contrast exposure within 1 week or less from the index procedure; (2) patients with a pre-existing end-stage renal disease requiring hemodialysis, estimated glomerular filtration rate (eGFR) < 45 mL/min/1.73m^2^; (3) no use of isotonic saline for hydration; (4) patients in shock; and (5) pregnant or lactating women.

CAG was performed according to the current guidelines using standard guide catheters, guidewires, balloon catheters, and stents via the femoral or radial approach. Serum creatinine concentrations were measured in all patients at hospital admission. The post-operative serum creatinine concentrations we used were the highest levels measured at least three times within a 72-h timeframe. The dose of contrast medium (CM) was calculated, and the interventional cardiologist was asked to minimize the dose of CM. The AHA/ACCF guidelines were followed regarding CM type and treatment process^17^. *Echocardiography was used to measure EF.* Demographics of the patients, clinical manifestations, laboratory results, angiographic and procedural characteristics were collected from the hospital database system.

### Definitions

HF is a clinical syndrome in which a series of symptoms and signs appear due to cardiac systolic or diastolic dysfunction, reducing cardiac output. HF was defined using the Framingham Heart Study (FHS) criteria, which require at least two major, or one major plus two minor criteria (Figure S1)^18^.

The left ventricular end-diastolic and end-systolic diameters (LVEDD and LVESD, respectively) in diastole were measured at the mid-papillary level on echocardiography two-dimensional short-axis pictures. EF was computed according to the classical *Teichholz* method^19^, EF = (LVEDV-LVESV)/LVEDV*100.

Based on the measurement of EF% guided by the 2016 European Society of Cardiology (ESC) guidelines, HF was classified as HFpEF if EF ≥ 50%; HFrEF if EF < 40%; and HFmrEF if EF in the range of 40–49%^14^. When HFpEF and HFmrEF were diagnosed, BNP > 35 ng/L or NT-proBNP > 125 ng/L needed to be met. An increase of either 25% or 0.5 mg/dL (44.2 μmoI/L) in serum basal creatinine level within 72 h following the administration of contrast agent was identified as CI-AKI^20^.

### Statistical analysis

Statistical analysis was performed using the SPSS statistical package, version 24.0 (Chicago, Illinois, USA). Categorical variables were expressed as numbers (proportions) and groups were compared using chi-square tests. Continuous variables were expressed as mean ± standard deviation (SD) or median and interquartile range, and groups were compared using non-parametric Mann–Whitney U test depending on the normal distribution of the sample.

Logistics regression analysis was used to identify the risk factors of CI-AKI among the whole population and patients with HF. Initially, in univariate analysis, variables with a *p* value < 0.1 were screened out, then we conducted multivariable-adjusted analysis that included the most promising variables. A Receiver Operating Characteristics (ROC) curve was created to estimate the predictive accuracy of the risk factors for CI-AKI development. Multivariate logistic regression and chi-square test were also performed in subgroup analysis. All reported *p* values were two-sided, and the *p* values < 0.05 were considered statistically significant.

### Ethics approval

The study was conducted according to the Declaration of Helsinki (as revised in 2013), and was approved by the ethics committee of Sir Run Run Shaw Hospital (NO.20201217-36).

## Results

### Baseline characteristics of all patients with or without CIAKI after coronary interventional diagnosis and treatment

A total of 3848 patients were enrolled. The mean age was 66.58 ± 10.71 years old, 33.9% females, 48.1% with HF, and 16.9% with CI-AKI. *Additionally, patients with New York Heart Association (NYHA) Grade II accounted for 33.5%, and Grade III accounted for 45.7%.*

Table 1 shows the baseline demographics, clinical and procedural characteristics of the whole patients. As shown, compared with patients without CI-AKI, those with CI-AKI were more likely to be older (68.7 ± 10.8 vs. 66.1 ± 10.6; *p* value < 0.001), female (39.8% vs. 32.7%; *p* value < 0.001), and having HF (55.5% vs. 46.6%; *p* value < 0.001), but less likely to receive renin-angiotensin system (RAS) inhibitors (39.3% vs. 44.9%, respectively; *p* value = 0.009). Also, CI-AKI patients, compared to those without, had higher levels of NT-proBNP, body mass index (BMI), C-reactive and protein (CRP), neutrophil to lymphocyte ratio (NLR), but lower levels of EF, hemoglobin and eGFR (*p* value for all < 0.05). No significant differences were observed in excess volumes of CM (CI-AKI: 1.8% vs. Non-CI-AKI: 1.9%, *p* value = 0.865), iso-osmolar CM (CI-AKI: 26.0% vs. Non-CI-AKI: 28.1%; *p* value = 0.287) and the use of diuretics (CI-AKI: 32.4% vs. Non-CI-AKI: 30.9%; *p* value = 0.449) between the two groups.

**Table 1:** Baseline clinical and procedural characteristics of all patients with and without CI-AKI.

Variable	Total (n = 3848)	CIAKI (n = 651)	Non-CIAKI (n = 3197)	*p* value
Age (years)	66.6 ± 10.7	68.7 ± 10.8	66.1 ± 10.6	< 0.001
Female, N (%)	1306(33.9%)	259(39.8%)	1047(32.7%)	< 0.001
BMI (kg/m^2^)	22.12 ± 8.7	22.87 ± 8.33	21.98 ± 8.77	0.015
Hypertension, N (%)	2360(61.3%)	401(61.6%)	1959(61.3%)	0.878
Diabetes, N (%)	859(22.3%)	163(25%)	696(21.8%)	0.068
HF, N (%)	1852(48.1%)	361(55.5%)	1491(46.6%)	< 0.001
Smoking, N (%)	676(17.6%)	102(15.7%)	574(18%)	0.162
Drinking, N (%)	618(16.1%)	90(13.8%)	528(16.5%)	0.088
CRP (mg/L)	9.56 ± 21.20	14.39 ± 27.61	8.58 ± 19.50	< 0.001
NLR	3.68 ± 4.46	5.11 ± 7.54	3.38 ± 3.44	< 0.001
Hemoglobin (g/dL)	12.87 ± 1.86	12.30 ± 2.12	13.02 ± 1.75	< 0.001
EF(%)	60.22 ± 12.85	56.95 ± 13.33	60.89 ± 12.65	< 0.001
NT-proBNP, pg/mL	1378.65 ± 2177.76	2357.52 ± 3098.51	1172.17 ± 1864.46	< 0.001
eGFR, mL/min/1.73m^2^	83.99 ± 16.82	84.78 ± 19.27	83.83 ± 16.28	0.253
Excess volumes of CM, N (%)	67(1.9%)	11(1.8%)	56(1.9%)	0.865
Iso-osmolar CM, N (%)	1063(27.7%)	169(26.0%)	894(28.1%)	0.287
Diuretics, N (%)	1199(31.2%)	211(32.4%)	988(30.9%)	0.449
RAS inhibitors, N (%)	1691(43.9%)	256(39.3%)	1435(44.9%)	0.009

Data are presented as mean ± SD, absolute n (%), or median (inter quartile range). The *p* values for continuous data were obtained from the analysis of the non-parametric Mann–Whitney U test, the *p* values for categorical data were obtained from the chi-square test. *CI-AKI* contrast-induced acute kidney injury; *BMI* body mass index; *HF* heart failure; *CRP* C-reactive protein; *NLR* neutrophil to lymphocyte ratio; *TC* total cholesterol; *LDL-C* low-density lipoprotein cholesterol; *EF* ejection fraction; *NT-proBNP* N-terminal pro b-type natriuretic peptide; *eGFR* estimated glomerular filtration rate; *CM* contrast medium; *RAS* renin-angiotensin system.

### Predictors of CI-AKI in the total study population

Through the univariate analysis, variables with *p* value < 0.1 were screened out for multivariate analyses. Multivariate logistic regression suggested that HF (adjusted odds ratio [OR] 1.316, 95% confidence interval [CI] 1.088–1.592, *p* value = 0.005), elderly (OR 1.021, 95% CI 1.011–1.031; *p* value < 0.001), female (OR 1.249, 95% CI 1.018–1.531, *p* value = 0.033), elevated levels of CRP (OR 1.009, 95% CI 1.005–1.013; *p* value < 0.001), and lower rate of use of RAS inhibitors (OR 0.813, 95% CI 0.670–0.987, *p* value = 0.037) were associated with increased risk of CI-AKI after CAG and PCI (Table 2).

**Table 2:** Univariate and multivariate logistic association for CI-AKI among the whole population.

Variable	Univariate regression			Multiple regression		
	OR	95% CI	*p*	OR	95% CI	*p*
Age (years)	1.024	1.016–1.033	< 0.001	1.021	1.011–1.031	< 0.001
Female, N (%)	1.357	1.141–1.614	0.001	1.249	1.018–1.531	0.033
BMI (kg/m^2^)	1.012	1.001–1.024	0.032	1.008	0.996–1.019	0.182
Hypertension, N (%)	1.014	0.852–1.205	0.878			
Diabetes, N (%)	1.200	0.986–1.537	0.068	1.155	0.925–1.442	0.204
HF, N (%)	1.424	1.202–1.687	< 0.001	1.316	1.088–1.592	0.005
Smoking, N (%)	0.849	0.675–1.068	0.163			
Drinking, N (%)	0.811	0.637–1.032	0.089	0.927	0.694–1.239	0.610
CRP (mg/L)	1.010	1.007–1.013	< 0.001	1.009	1.005–1.013	< 0.001
eGFR, mL/min/1.73m^2^	1.003	0.998–1.008	0.191			
Excess volumes of CM, N (%)	0.945	0.492–1.814	0.865			
Iso–osmolar CM, N (%)	0.901	0.744–1.091	0.287			
Diuretics, N (%)	1.072	0.895–1.284	0.449			
RAS inhibitors, N (%)	0.796	0.670–0.945	0.009	0.813	0.670–0.987	0.037

Values are expressed as mean ± SD or n (%) unless otherwise indicated. OR,odds ratio; CI, confidence interval. Other abbreviations as in Table 1.

### Predictors of CI-AKI among patients with HF

Multivariate logistic regression showed that elderly (OR 1.017, 95% CI 1.005–1.030; *p* value = 0.004), female (OR 1.739, 95% CI 1.351–2.238; *p* value < 0.001), lower levels of EF (OR 0.985, 95% CI 0.976–0.994, *p* value = 0.001), elevated levels of NT-proBNP (OR 1.168, 95% CI 1.118–1.222; *p* value < 0.001) and CRP (OR 1.008, 95% CI 1.003–1.012; *p* value = 0.001), and low rate of use of RAS inhibitors (OR 0.698, 95% CI 0.545–0.893, *p* value = 0.004) were associated with increased risk of CI-AKI among patients with HF (Table 3).

**Table 3:** Univariate and multivariate logistic association for CI-AKI among patients with HF.

Variable	Univariate Regression			Multiple Regression		
	OR	95% CI	*p*	OR	95% CI	*p*
Age (years)	1.026	1.015–1.038	< 0.001	1.017	1.005–1.030	0.004
Female, N (%)	1.647	1.301–2.084	< 0.001	1.739	1.351–2.238	< 0.001
BMI (kg/m^2^)	1.011	0.995–1.026	0.169			
Hypertension, N (%)	1.061	0.839–1.341	0.621			
Diabetes, N (%)	1.182	0.904–1.546	0.221			
Smoking, N (%)	0.872	0.640–1.186	0.382			
Drinking, N (%)	0.848	0.608–1.182	0.330			
CRP (mg/L)	1.010	1.006–1.014	< 0.001	1.008	1.003–1.012	0.001
eGFR, mL/min/1.73m^2^	0.997	0.991–1.004	0.428			
EF(%)	0.982	0.974–0.990	< 0.001	0.985	0.976–0.994	0.001
NT–proBNP, pg/mL	1.220	1.170–1.272	< 0.001	1.168	1.118–1.222	< 0.001
Excess volumes of CM, N (%)	0.537	0.188–1.535	0.246			
Iso–osmolar CM, N (%)	0.980	0.764–1.258	0.875			
Diuretics, N (%)	1.229	0.964–1.568	0.097			
RAS inhibitors, N (%)	0.715	0.566–0.904	0.005	0.698	0.545–0.893	0.004

Values are expressed as mean ± SD or n (%) unless otherwise indicated. OR,odds ratio; CI, confidence interval. Other abbreviations as in Table 1.

**Figure 2:**
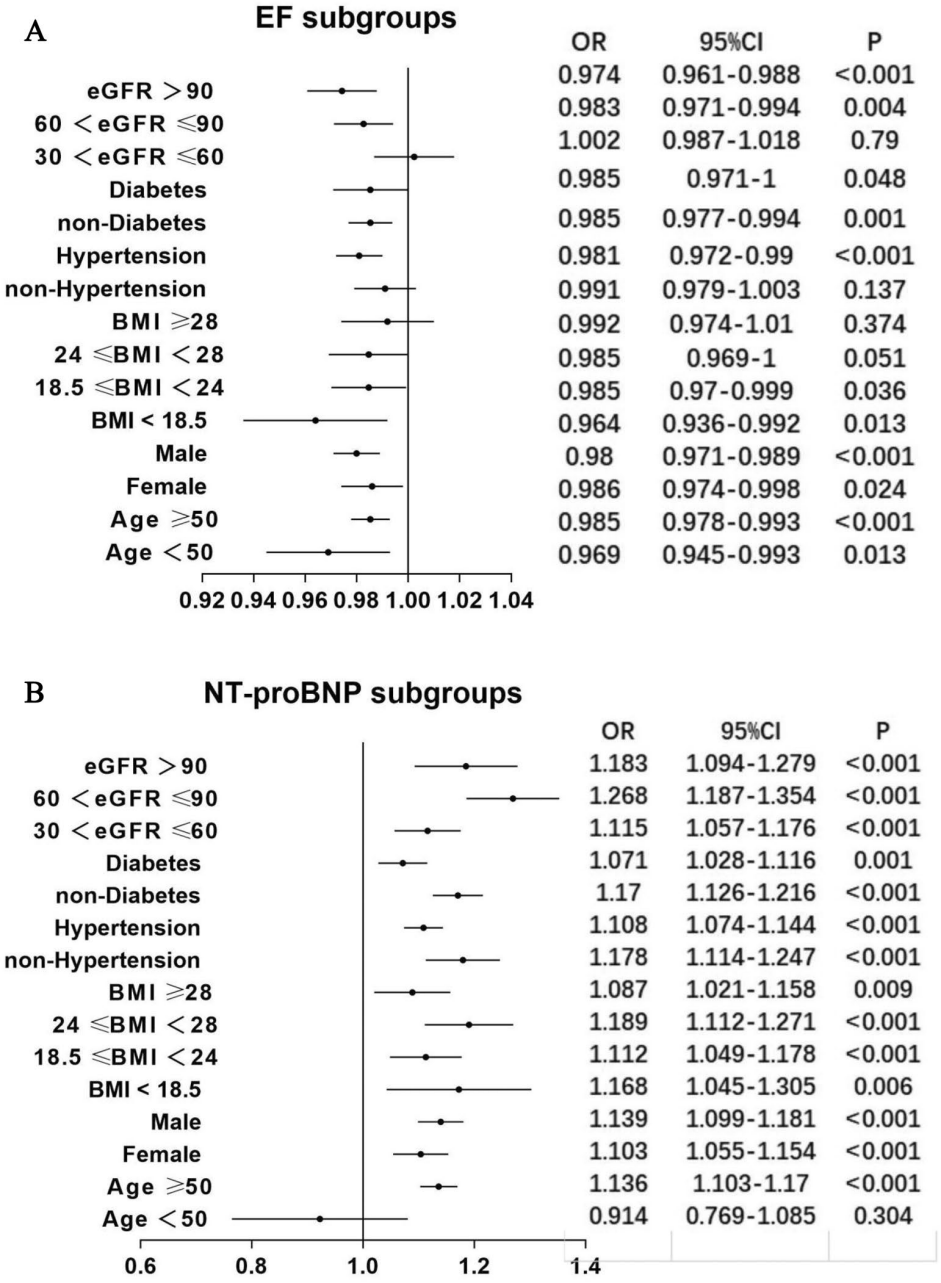
(**A**) The relationship between EF and CI-AKI in patients with HF was analysed by multivariable logistic regression analysis in predefined subgroups. (**B**) The relationship between NT-proBNP and CI-AKI in patients with HF was analysed by multivariable logistic regression analysis in predefined subgroups. *NT-proBNP* N-terminal pro b-type natriuretic peptide; *BMI* body mass index; *eGFR* estimated glomerular filtration rate; *OR* odds ratio; *CI* confidence interval. Other abbreviations as in Fig. 1.

To further investigate the relationship between EF, NT-proBNP and CI-AKI among patients with HF, we conducted a subgroup analysis. The results were consistent in most subgroups, as shown in Fig. 2. The subgroup analysis also suggests that the correlation between NT-proBNP (Fig. 2B) and CI-AKI was more stable than the correlation between EF (Fig. 2A) and CI-AKI.

**Figure 3:**
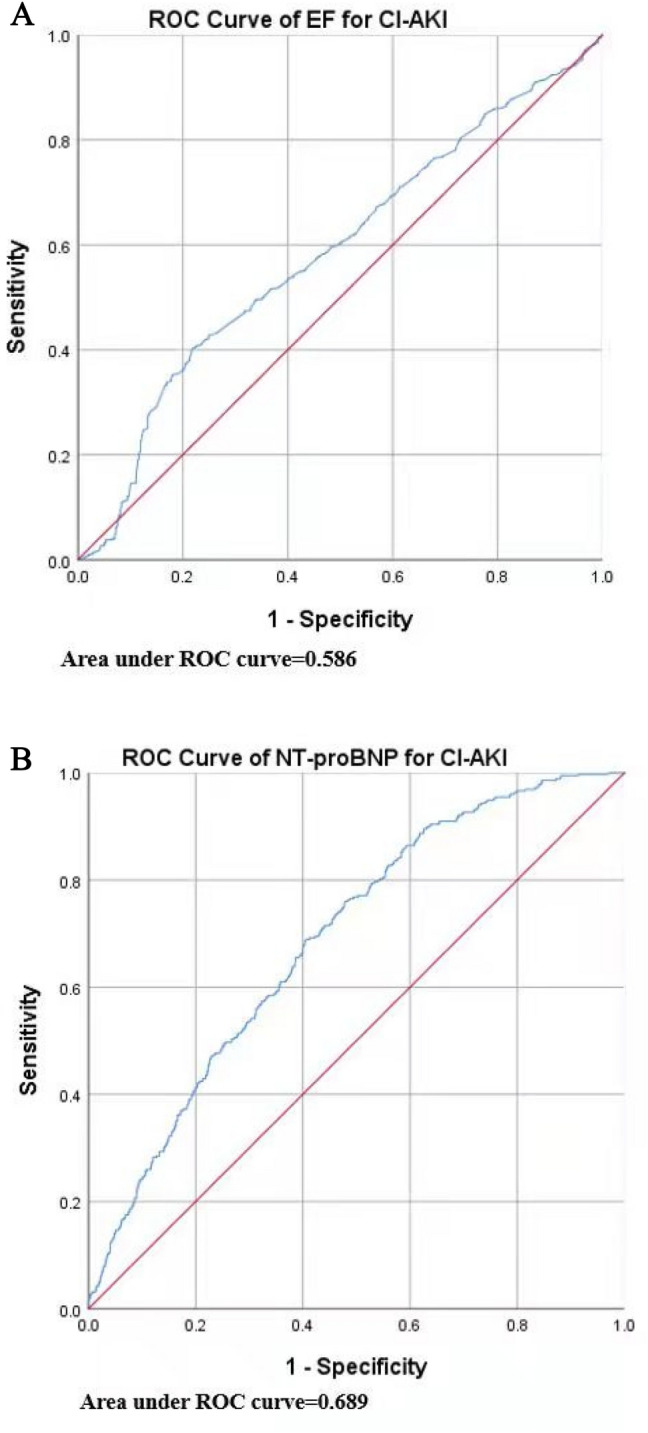
(**A**) The cutoff value of EF for predicting CI-AKI in patients with HF was analysed by ROC curve. (**B**) The cutoff value of NT-proBNP for predicting CIAKI in patients with HF was analysed by ROC curve. NT-proBNP, N-terminal pro b-type natriuretic peptide. Other abbreviations as in Fig. 1.

For optimal prediction of CI-AKI among patients with HF, we used the ROC curve to identify the ideal cut-off predictive values of EF and NT-proBNP (Fig. 3A,B). The best predictive cut-off value of EF was 61.15%, and for NT-proBNP it was 1773 ng/L.

### Association of EF, NT-proBNP and CI-AKI in the HFrEF, HFmrEF and HFpEF groups

Table 4 shows the associations between the classification of HF and CI-AKI. As shown, patients with HFrEF were more likely to develop CI-AKI than those with HFmrEF or HFpEF (OR 0.852, 95% CI 0.738–0.985; *p* value = 0.031).

**Table 4:** Univariate and multivariate logistic association to investigate the relationship between the classification of HF and CI-AKI.

Variable	Univariate Regression			Multiple Regression		
	OR	95% CI	*p*	OR	95% CI	*p*
Age (years)	1.026	1.015–1.038	< 0.001	1.024	1.012–1.037	< 0.001
Female, N (%)	1.647	1.301–2.084	< 0.001	1.691	1.301–2.199	< 0.001
BMI (kg/m^2^)	1.011	0.995–1.026	0.169			
Hypertension, N (%)	1.061	0.839–1.341	0.621			
Diabetes, N (%)	1.182	0.904–1.546	0.221			
Smoking, N (%)	0.872	0.640–1.186	0.382			
Drinking, N (%)	0.848	0.608–1.182	0.330			
CRP (mg/L)	1.010	1.006–1.014	< 0.001	1.009	1.005–1.014	< 0.001
eGFR, mL/min/1.73m^2^	0.997	0.991–1.004	0.428			
Excess volumes of CM, N (%)	0.537	0.188–1.535	0.246			
Iso–osmolar CM, N (%)	0.980	0.764–1.258	0.875			
Different categories of HF, N (%)	0.857	0.743–0.988	0.033	0.838	0.719–0.977	0.024
Diuretics, N (%)	1.229	0.964–1.568	0.097			
RAS inhibitors, N (%)	0.715	0.566–0.904	0.005	0.704	0.554–0.895	0.004

Values are expressed as mean ± SD or n (%) unless otherwise indicated. OR,odds ratio; CI, confidence interval. Other abbreviations as in Table 1.

**Figure 4:**
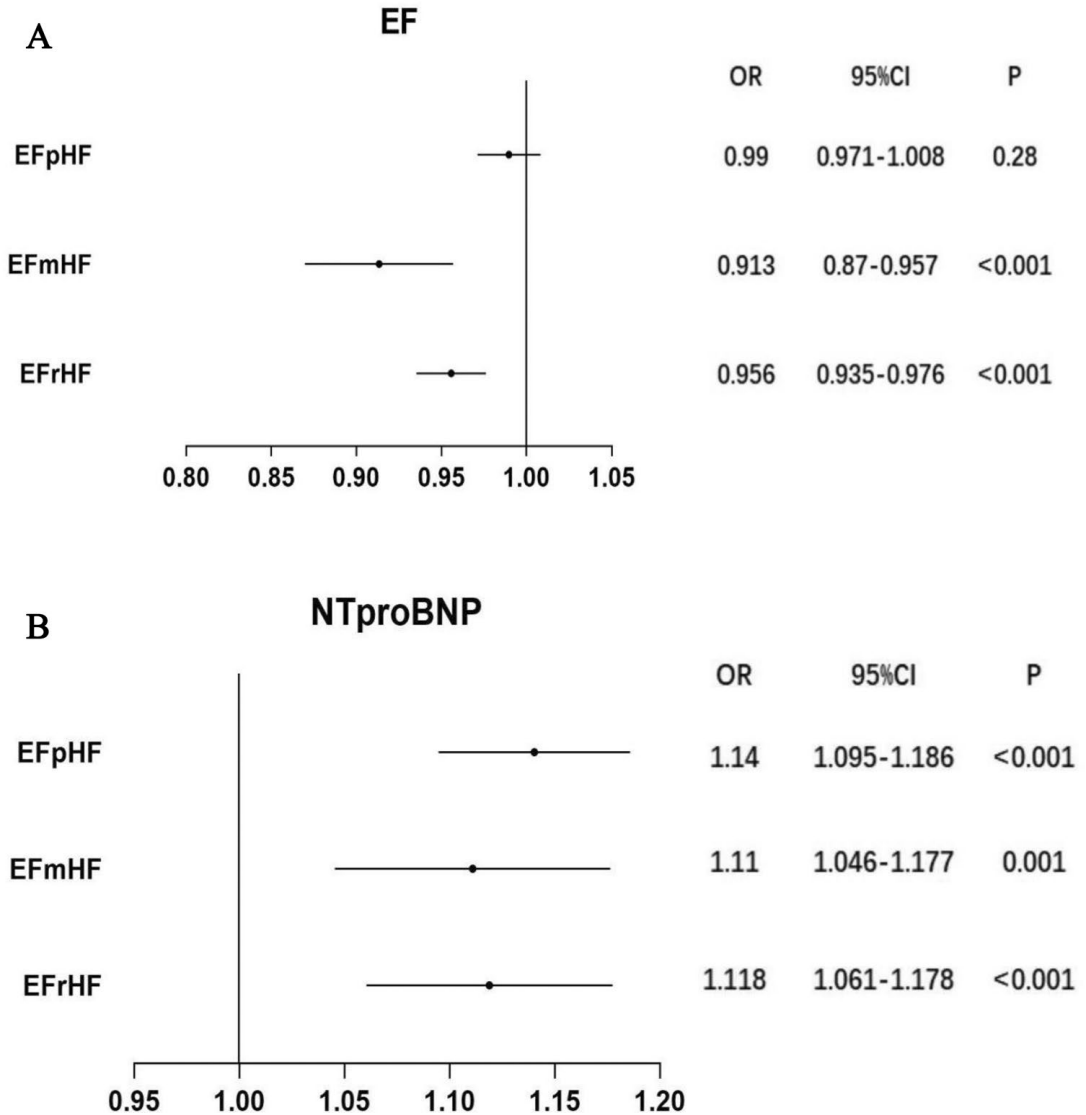
(**A**) The relationship between EF and CI-AKI was analysed by multivariable logistic regression analysis in HFrEF, HFmrEF and HFpEF groups. (**B**) The relationship between NT-proBNP and CI-AKI was analysed by multivariable logistic regression analysis in HFrEF, HFmrEF and HFpEF groups. *NT-proBNP* N-terminal pro b-type natriuretic peptide; *OR* odds ratio; *CI* confidence interval. Other abbreviations as in Fig. 1.

Subgroup analysis based on the classification of HF suggested that lower levels of EF were risk factors for CI-AKI in the HFrEF (OR 0.956, 95% CI 0.935–0.976; *p* value < 0.001) and HFmrEF (OR 0.913, 95% CI 0.870–0.957, *p* value < 0.001) groups, but not in the HFpEF group. Elevated levels of NT-proBNP were an independent risk factor for CI-AKI in the HFrEF (OR 1.118, 95% CI 1.061–1.178; *p* value < 0.001), HFmrEF (OR 1.110, 95% CI 1.046–1.177; *p* value = 0.001) and HFpEF (OR 1.140, 95% CI 1.095–1.186, *p* < 0.001) groups (Fig. 4).

## Discussion

The pathophysiological process of CI-AKI is very complex and remains poorly understood. Nevertheless, it is well recognized that hemodynamic deterioration plays a vital role in the development of CI-AKI^20,21^. Deterioration of cardiac function contributes to the hemodynamic instability, which can decrease the effective renal perfusion pressure, consequently activating the renin-angiotensin system and sympathetic nervous system, increasing inflammatory factors and oxygen radical levels, all of which contribute to the development of CI-AKI^22^. The Mehran risk score is a classical risk assessment model for CI-AKI^9^. Of its eight variables, three (hypotension, advanced HF and use of intra-aortic balloon pump) directly reflect worsened cardiac function^9^. A prior report from a randomized trial carried out among 142 patients suggested that HF is correlated with CI-AKI incidence^23^. Consistent with these findings, we observed a significant association between HF and CI-AKI in our multivariable logistic regression model.

After confirming that HF is an independent risk factor for CI-AKI in the whole population, we examined risk factors for CI-AKI in the HF population. The results showed that the classification of HF according to EF was related to the incidence of CI-AKI. After adjusting for several major confounders, the current study confirmed that the classification and severity of HF are independent risk factors for CI-AKI among patients with HF.

EF is the most widely used parameter to evaluate cardiac functions. Low EF is associated with hemodynamic instability, and consequently causes inadequate renal perfusion^24^. However, the association of EF with CI-AKI remains controversial. A recent study including 138 patients with acute myocardial infarction found that patients with low EF had a significantly higher rate of CI-AKI after the second step of staged coronary revascularization for acute myocardial infarction^25^. An observational study by Shacham et al. showed that worsening EF was an independent predictor of CI-AKI^26^. On the other hand, Barbieri et al.^27^ and Kurtul et al.^28^ showed an opposite effect after adjusting for several confounders. Another single-center prospective observational study compared the incidence of CI-AKI among different categories of HF^16^. Results from these studies showed that there were no significant differences between HFpEF, HFrEF and HFmrEF groups^16^. Our study included sufficient patients with HF and adjusted for the potential confounders, which overcome some of the limitations in previous studies.

Our study supports the idea that EF is an independent predictor of CI-AKI among patients with HF. Furthermore, we showed that EF was negatively correlated with CI-AKI incidence in HFrEF and HFmrEF groups. In recent years, the number of patients with HFpEF has been increasing significantly^29^. The diagnosis of HFpEF is more challenging than the diagnosis of HFrEF, which might cause patients with HFpEF to receive less attention in clinical practice^30^. However, we found no significant association between EF and CI-AKI in the HFpEF group for better or worse. In other words, when EF ≥ 50%, we do not need to pay too much attention to the accurate values of EF to prevent CI-AKI.

NT-proBNP is another valuable biomarker that is used extensively to monitor the severity of cardiac dysfunction^31^. A previous study confirmed that B-type natriuretic peptide (BNP) levels measured on admission were associated with acute kidney injury and its severity in patients hospitalized with acute coronary syndromes^32^. Although BNP levels correlate with NT-proBNP levels, NT-proBNP levels are more sensitive and stable than BNP levels because of a longer half-life^33^. Elevated levels of NT-proBNP reflect hemodynamic instability, myocardial ischemia and reduced renal perfusion, all contribute to the development of CI-AKI through complex physiological and pathological alterations^33^. Few studies have examined the ideal NT-proBNP cut-off value for predicting CI-AKI. We filled this knowledge gap and provided a cut-off point (≥ 1773 pg/mL) for NT-proBNP that best predict CI-AKI among patients with HF patients.

Wang et al. showed significant associations between NT-proBNP levels with CI-AKI and long-term mortality in patients with HFmrEF undergoing CAG/PCI^34^. Our study further confirmed that NT-proBNP levels were an independent risk factor for CI-AKI in the HFrEF, HFmrEF and HFpEF groups. This could partially explain that HF in the deterioration period is an independent risk factor for CI-AKI. Thus, NT-proBNP appears to be a promising preoperative biomarker of CI-AKI risk in patients with HF undergoing CAG/PCI.

Strengths of our study include its multicenter nature and large sample size. Also, to the best of our knowledge, few studies have been conducted to explore the predictors of CI-AKI after coronary interventional diagnosis and treatment focusing on patients with HF. Additionally, the study further investigated the association of EF and NT-proBNP with CIAKI in HFpEF, HFrEF and HFmrEF group, separately. Thus, findings from our study could potentially help in early identification of HF patients at high risk of CI-AKI who may benefit from close monitoring.

Our study also has some limitations that deserve discussion. First, as, retrospective observational study, the risk of bias and residual confounding cannot be completely ruled out, although we attempted to adjust for the confounding factors. Therefore, large-scale randomized controlled trials are needed before these conclusions are to be applied elsewhere. Second, one of the inclusion criteria is that patients with documented serum creatinine before and 72 h after CAG/PCI. Patients with elevated baseline serum creatinine levels may be more inclined to measure serum creatinine at 72 h after CAG/PCI. This might have resulted in selection bias. Third, most patients with stable conditions after coronary intervention were discharged from the hospital the next day, which caused a higher incidence of HF in the data analysis. However, this did not impact our ability to explore CI-AKI in patients with HF. Fourth, we did not have information on the amount of fluid infusion used during the procedures. Hence, we were not able to adjust for that variable in the models. However, each patient undergoing CAG and PCI was hydrated according to the guidelines at the time. Finally, diagnosis of HF based on clinical symptoms and signs was relatively subjective, which has limited reliability.

## Conclusions

Elevated levels of NT-proBNP are independent risk factors for CI-AKI irrespective of the classification of HF. Decreased levels of EF were risk factors for CI-AKI in the HFrEF and HFmrEF groups, but not in the HFpEF group.

## Supplementary Information

Supplementary Information.

## Data Availability

Statistical analysis was performed using the SPSS statistical package, version 24.0 (Chicago, Illinois, USA).

## References

[1] Wang C, Chen W, Yu M, Yang P (2020). Comparison of acute kidney injury with radial vs. femoral access for patients undergoing coronary catheterization: an updated meta-analysis of 46,816 patients. Exp. Ther. Med..

[2] Rear R, Bell RM, Hausenloy DJ (2016). Contrast-induced nephropathy following angiography and cardiac interventions. Heart.

[3] Stub D, Lauck S, Lee M, Gao M, Humphries K, Chan A (2015). Regional systems of care to optimize outcomes in patients undergoing transcatheter aortic valve replacement. JACC Cardiovasc. Interv..

[4] Chalikias G, Drosos I, Tziakas DN (2016). Contrast-induced acute kidney injury: an update. Cardiovasc. Drugs Ther..

[5] Azzalini L, Kalra S (2020). Contrast-induced acute kidney injury-definitions, epidemiology, and implications. Interv. Cardiol. Clin..

[6] McCullough PA, Choi JP, Feghali GA, Schussler JM, Stoler RM, Vallabahn RC (2016). Contrast-induced acute kidney injury. J. Am. Coll. Cardiol..

[7] Stacul F, van der Molen AJ, Reimer P, Webb JA, Thomsen HS, Morcos SK (2011). Contrast induced nephropathy: updated ESUR contrast media safety committee guidelines. Eur. Radiol..

[8] Duan C, Cao Y, Liu Y, Zhou L, Ping K, Tan MT (2017). A New Preprocedure risk score for predicting contrast-induced acute kidney injury. Can. J. Cardiol..

[9] Mehran R, Aymong ED, Nikolsky E, Lasic Z, Iakovou I, Fahy M (2004). A simple risk score for prediction of contrast-induced nephropathy after percutaneous coronary intervention: development and initial validation. J. Am. Coll. Cardiol..

[10] Conrad N, Judge A, Tran J, Mohseni H, Hedgecott D, Crespillo AP (2018). Temporal trends and patterns in heart failure incidence: a population-based study of 4 million individuals. Lancet.

[11] Bei WJ, Wang K, Li HL, Guo XS, Guo W, Abuduaini T (2019). Safe hydration to prevent contrast-induced acute kidney injury and worsening heart failure in patients with renal insufficiency and heart failure undergoing coronary angiography or percutaneous coronary intervention. Int. Heart J..

[12] Feldkamp T, Luedemann M, Spehlmann ME, Freitag-Wolf S, Gaensbacher J, Schulte K (2018). Radial access protects from contrast media induced nephropathy after cardiac catheterization procedures. Clin. Res. Cardiol..

[13] Chen SQ, Liu Y, Bei WJ, Wang Y, Duan CY, Wu DX (2018). Optimal hydration volume among high-risk patients with advanced congestive heart failure undergoing coronary angiography. Oncotarget.

[14] Ponikowski P, Voors AA, Anker SD, Bueno H, Cleland JGF, Coats AJS (2016). 2016 ESC guidelines for the diagnosis and treatment of acute and chronic heart failure: the task force for the diagnosis and treatment of acute and chronic heart failure of the European Society of Cardiology (ESC)Developed with the special contribution of the Heart Failure Association (HFA) of the ESC. Eur. Heart J..

[15] Roberts E, Ludman AJ, Dworzynski K, Al-Mohammad A, Cowie MR, McMurray JJ (2015). The diagnostic accuracy of the natriuretic peptides in heart failure: systematic review and diagnostic meta-analysis in the acute care setting. BMJ.

[16] Wang K, Li HL, Bei WJ, Guo XS, Chen SQ, Islam SMS (2017). Association of left ventricular ejection fraction with contrast-induced nephropathy and mortality following coronary angiography or intervention in patients with heart failure. Ther. Clin. Risk Manag..

[17] Wagner GS, Macfarlane P, Wellens H, Josephson M, Gorgels A, Mirvis DM (2009). AHA/ACCF/HRS recommendations for the standardization and interpretation of the electrocardiogram: part VI: acute ischemia/infarction: a scientific statement from the american heart association electrocardiography and arrhythmias committee, council on clinical cardiology; the American College of Cardiology Foundation; and the Heart Rhythm Society: endorsed by the International Society for Computerized Electrocardiology. Circulation.

[18] Andersson C, Vasan RS (2014). Epidemiology of heart failure with preserved ejection fraction. Heart Fail. Clin..

[19] Teichholz LE, Kreulen T, Herman MV, Gorlin R (1976). Problems in echocardiographic volume determinations: echocardiographic-angiographic correlations in the presence of absence of asynergy. Am. J. Cardiol..

[20] Fahling M, Seeliger E, Patzak A, Persson PB (2017). Understanding and preventing contrast-induced acute kidney injury. Nat. Rev. Nephrol..

[21] Vlachopanos G, Schizas D, Hasemaki N, Georgalis A (2019). Pathophysiology of contrast-induced acute kidney injury (CIAKI). Curr. Pharm. Des..

[22] Azzalini L, Spagnoli V, Ly HQ (2016). Contrast-induced nephropathy: from pathophysiology to preventive strategies. Can. J. Cardiol..

[23] Rosenstock JL, Gilles E, Geller AB, Panagopoulos G, Mathew S, Malieckal D (2010). Impact of heart failure on the incidence of contrast-induced nephropathy in patients with chronic kidney disease. Int. Urol. Nephrol..

[24] Chioncel O, Lainscak M, Seferovic PM, Anker SD, Crespo-Leiro MG, Harjola VP (2017). Epidemiology and one-year outcomes in patients with chronic heart failure and preserved, mid-range and reduced ejection fraction: an analysis of the ESC heart failure long-term registry. Eur. J. Heart Fail..

[25] Chyrchel M, Halubiec P, Lazarczyk A, Duchnevic O, Okarski M, Gebska M (2020). Low ejection fraction predisposes to contrast-induced nephropathy after the second step of staged coronary revascularization for acute myocardial infarction: a retrospective observational study. J. Clin. Med..

[26] Shacham Y, Leshem-Rubinow E, Gal-Oz A, Topilsky Y, Steinvil A, Keren G (2015). Association of left ventricular function and acute kidney injury among ST-elevation myocardial infarction patients treated by primary percutaneous intervention. Am. J. Cardiol..

[27] Barbieri L, Verdoia M, Nardin M, Marino P, Suryapranata H, De Luca G (2017). Gender difference in the risk of contrast-induced nephropathy in patients undergoing coronary angiography or percutaneous coronary intervention. Angiology.

[28] Kurtul A, Duran M, Yarlioglues M, Murat SN, Demircelik MB, Ergun G (2014). Association between N-terminal pro-brain natriuretic peptide levels and contrast-induced nephropathy in patients undergoing percutaneous coronary intervention for acute coronary syndrome. Clin. Cardiol..

[29] Redfield MM (2016). Heart failure with preserved ejection fraction. N. Engl. J. Med..

[30] Gladden JD, Chaanine AH, Redfield MM (2018). Heart failure with preserved ejection fraction. Annu. Rev. Med..

[31] McKie PM, Burnett JC (2016). NT-proBNP: the gold standard biomarker in heart failure. J. Am. Coll. Cardiol..

[32] Moltrasio M, Cabiati A, Milazzo V, Rubino M, De Metrio M, Discacciati A (2014). B-type natriuretic peptide and risk of acute kidney injury in patients hospitalized with acute coronary syndromes*. Crit. Care Med..

[33] Stokes NR, Dietz BW, Liang JJ (2016). Cardiopulmonary laboratory biomarkers in the evaluation of acute dyspnea. Open Access. Emerg. Med..

[34] Wang K, Li HL, Chen LL, Bei WJ, Lin KY, Smyth B (2017). Association of N-terminal pro-brain natriuretic peptide with contrast-induced acute kidney injury and long-term mortality in patients with heart failure and mid-range ejection fraction: an observation study. Medicine (Baltimore).

