# Supplementary material for: Association of the classification and severity of heart failure with the incidence of contrast-induced acute kidney injury
Source: Sci Rep. 2021 Jul 28;11:15348. doi: 10.1038/s41598-021-94910-1 (PMC8319404; doi:10.1038/s41598-021-94910-1)
Supplement: Supplementary file 1 — Supplementary Information. [file 41598_2021_94910_MOESM1_ESM.docx]

Figure S1. Framingham Heart Study (FHS) Criteria.


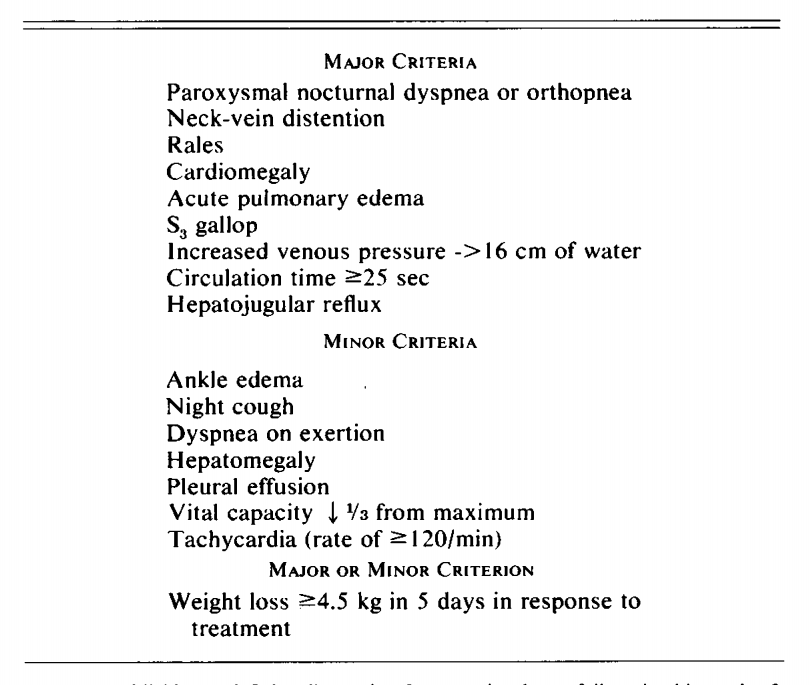


Heart Failure is defined that 2 major or 1 major ＆ 2 minor criteria have to be present concurrently.
